# A Digital Image Analysis of the Morphology and Immunolocalization of Catalase and Caspase-3 in the Skin of Adult Male Rats After Treatment with Letrozole and Vitamin C

**DOI:** 10.3390/ijms26178645

**Published:** 2025-09-05

**Authors:** Anna Pilutin, Julia Łukasiewicz, Sylwia Rzeszotek, Kamila Misiakiewicz-Has, Aleksandra Wilk

**Affiliations:** Department of Histology and Embryology, Pomeranian Medical University, Powstańców Wlkp. 72 Str., 70-111 Szczecin, Poland; julia.lukasiewicz1998@gmail.com (J.Ł.); sylwia.rzeszotek@pum.edu.pl (S.R.); kamila.misiakiewicz.has@pum.edu.pl (K.M.-H.); aleksandra.wilk@pum.edu.pl (A.W.)

**Keywords:** estrogens, male, skin, aromatase, letrozole, morphology, apoptosis, oxidative stress, artificial intelligence

## Abstract

Estrogens are hormones that play an important role in the skin, including in men. Letrozole (LET) is an inhibitor of the enzyme that converts androgens to estrogens. The use of letrozole can cause morphological changes and changes in the immunoexpression of proteins associated with oxidative stress and apoptosis. Vitamin C is a factor that modulates cellular stress. The purpose of this study was to examine whether letrozole and/or vitamin C supplementation can affect the morphology of the skin, parameters of the programmed cell death marker, and oxidative damage. Three-month-old rats were divided into four groups and treated with: (I) CTRL—water; (II) VIT C—L-ascorbic acid; (III) LET—letrozole; and (IV) LET+C—letrozole + L-ascorbic acid. The morphometrical measurements included epithelial thickness, width of collagen fibers, and elastic fibers. The expression levels of caspase-3 and catalase were determined. Significant differences in the morphometrical measurements and immunoexpression were observed. The findings indicate that chronic treatment with letrozole can affect morphology and induce oxidative stress and programmed cell death in the epidermal cells of adult male rats. Vitamin C supplementation exerts an effect on some parameters of the molecular processes.

## 1. Introduction

Skin is a tissue controlled by estrogens. It is known that in addition to being the target of these hormones, it is also considered the site of their production [1,2,3]. The enzyme responsible for converting androgens to estrogens is cytochrome P450 aromatase (P450arom). In humans, this enzyme is encoded by the *CYP19A1* gene [4]. Aromatization is an irreversible reaction, and there is no alternative pathway for androgen conversion. Therefore, the interruption or weakening of aromatase activity may lead to the cessation of estrogen formation [5,6]. Aromatase expression in the skin occurs mainly in hair follicles and sebaceous glands. P450arom synthesis by skin fibroblasts may be responsible for a significant part of peripheral estrogen synthesis, especially in postmenopausal women. Adipose tissue also plays an important role, as adipocytes present in dermal tissue also demonstrate the ability to aromatize androgens to estrogens [7]. Furthermore, aromatase activity is also demonstrated in hair follicle cells and melanocytes [8]. Estrogens exert their biological function through two types of estrogen receptors (ER): ERα and ERβ. In human skin, a greater expression of ERβ is observed compared to ERα [9]. The presence of ERα is demonstrated by sebaceous gland cells, whereas the expression of ERβ is found in epidermal cells, sebaceous and sweat gland cells, as well as in hair follicle cells [1]. Due to the wide distribution of estrogen receptors in the skin, these hormones play a significant role in processes such as skin pigmentation, hair growth, sebum production, and skin cancer [10]. Moreover, estrogens play a pleiotropic role in skin aging [3]. These hormones increase blood flow, skin thickness, and moisture levels by promoting the production of hyaluronic acid, mucopolysaccharides, and sebum. Estrogen positively influences the survival of skin fibroblasts. By promoting the production of transforming growth factor-beta (TGFβ) and tissue inhibitors of matrix metalloproteinases (TIMPs) while also suppressing matrix metalloproteinases (MMPs), estrogens encourage the synthesis and preservation of extracellular matrix elements like collagen types I and III and fibrillin [11].

Letrozole (LET) is a nonsteroidal inhibitor of cytochrome P450 aromatase, an enzyme that converts androgens to estrogens [12]. It is widely used in clinical practice [13], among others, in the treatment of breast cancer [14] and endometriosis [4]. In men, it has been used in the treatment of infertility [14] and hypogonadism [15]. It has also been used in boys with diagnosed idiopathic short stature [16] and constitutional delayed puberty [17]. Treatment with anti-estrogen drugs, such as tamoxifen or aromatase inhibitors, is also associated with inadequate wound healing [3].

An important factor affecting normal skin physiology is vitamin C. It promotes collagen biosynthesis, inhibits melanogenesis, prevents radiation damage, accelerates the wound healing process [18,19,20,21], and downregulates the MMPs responsible for collagen degradation [22]. Topical administration of ascorbic acid in dermatology helps burn wounds heal in two ways: it promotes the formation of collagen in skin tissues and acts as an antioxidant by removing free radicals [23]. Vitamin C is one of the most important antioxidants and can be considered the “central element” of the antioxidant system. It reacts and inactivates free radicals in the plasma, cytosol, and extracellular fluid compartments of the body. Vitamin C can regenerate vitamin E in its oxidized form. The functionality of these processes is facilitated by their capacity to donate electrons [24]. As the ascorbyl radical is relatively stable and easily regenerated back to ascorbic acid, vitamin C can participate in antioxidant reactions multiple times [25].

Catalase expression is an important skin protective mechanism that prevents lipid peroxidation and DNA and protein damage, which could lead to premature skin aging or cancerous lesions [26]. Catalase activity may decrease with age, which weakens the skin’s ability to neutralize oxidative stress [27].

Apoptosis determines the maintenance of homeostasis within the epidermis, as well as the functioning of the immune system [28]. Caspase-3 plays an important role in apoptosis processes. Its expression occurs in epidermal cells, especially when apoptosis is induced by stressors [29].

Earlier studies conducted by our team have shown that the long-term administration of LET to male rats led to morphological changes similar to the aging characteristics of the gonads [30,31], epididymis [32], bone tissue [33], and small intestine [34]. Our study aimed to investigate whether LET and LET, administered in combination with vitamin C, can affect the morphology and morphometry of the skin. In addition, pilot studies were conducted to determine whether LET and LET administered in combination with vitamin C can change certain parameters of programmed cell death markers and oxidative damage.

## 2. Results

### 2.1. Body Weight

We observed a decrease in body weight in both groups of animals treated with the aromatase inhibitor, but these changes were not statistically significant.

### 2.2. Morphology

**Figure 1:**
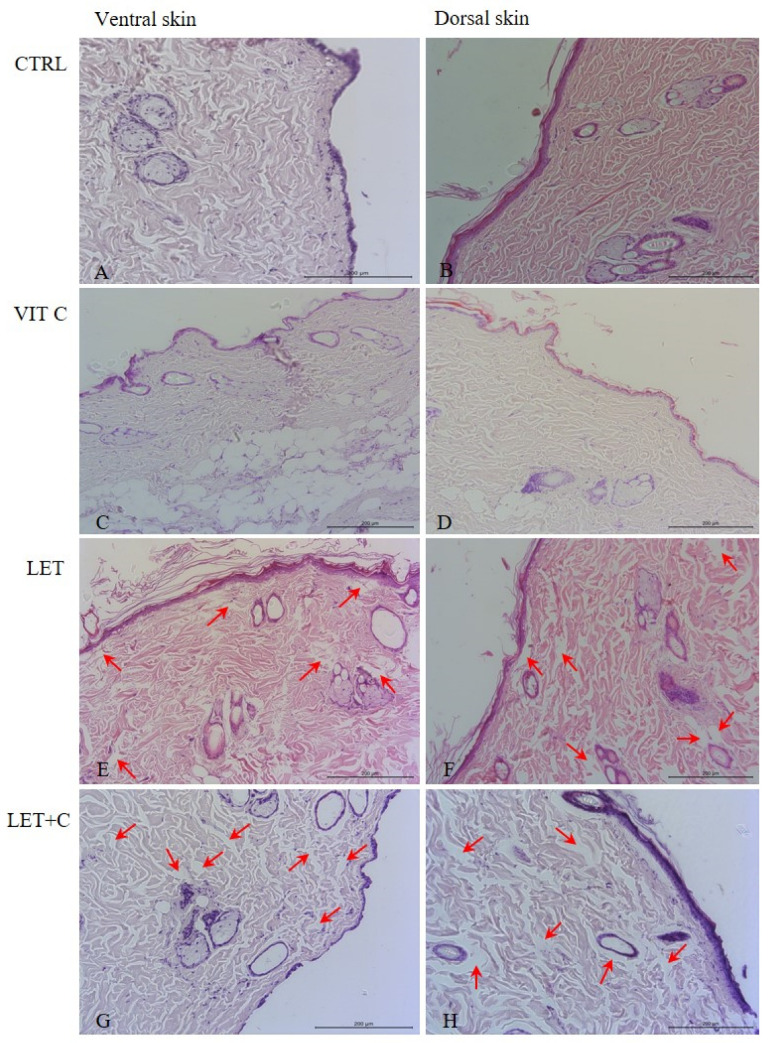
Organization of collagen fibers in the ventral and dorsal dermis. Empty spaces between collagen fibers (red arrows). (**A**,**B**)—control group (CTRL); (**C**,**D**)—group supplemented with vitamin C (VIT C); (**E**,**F**)—letrozole-treated group (LET); (**G**,**H**)—letrozole-treated and vitamin C-supplemented group (LET+C); HE, objective magnification ×20.

In both the ventral and dorsal skin, the collagen fiber bundles were correctly arranged in the CTRL (Figure 1A,B) and VIT C (Figure 1C,D) groups. In the papillary layer, collagen fiber bundles were arranged parallel to the epithelial surface. Flattened fibroblast cell nuclei and the nuclei of other connective tissue cells were observed between the collagen fibers. Collagen fiber bundles in the reticular layer formed a spatial network composed of tightly packed thick bundles running in different directions. Cell nuclei of fibroblasts and other connective tissue cells were visible between the fiber bundles.

Visible changes in the morphology of the ventral (Figure 1E) and dorsal (Figure 1F) skin in the LET group were observed. The distribution of collagen fiber bundles in both layers of the dermis was similar to that in the CTRL group, but empty spaces between fibers were observed in the LET group (red arrows).

The distribution of collagen fiber bundles in both layers of the dermis from the ventral (Figure 1G) and dorsal (Figure 1H) parts in the LET+C group was similar to that in the CTRL. However, empty spaces between fibers were observed (red arrows), similar to those in the LET group.

**Figure 2:**
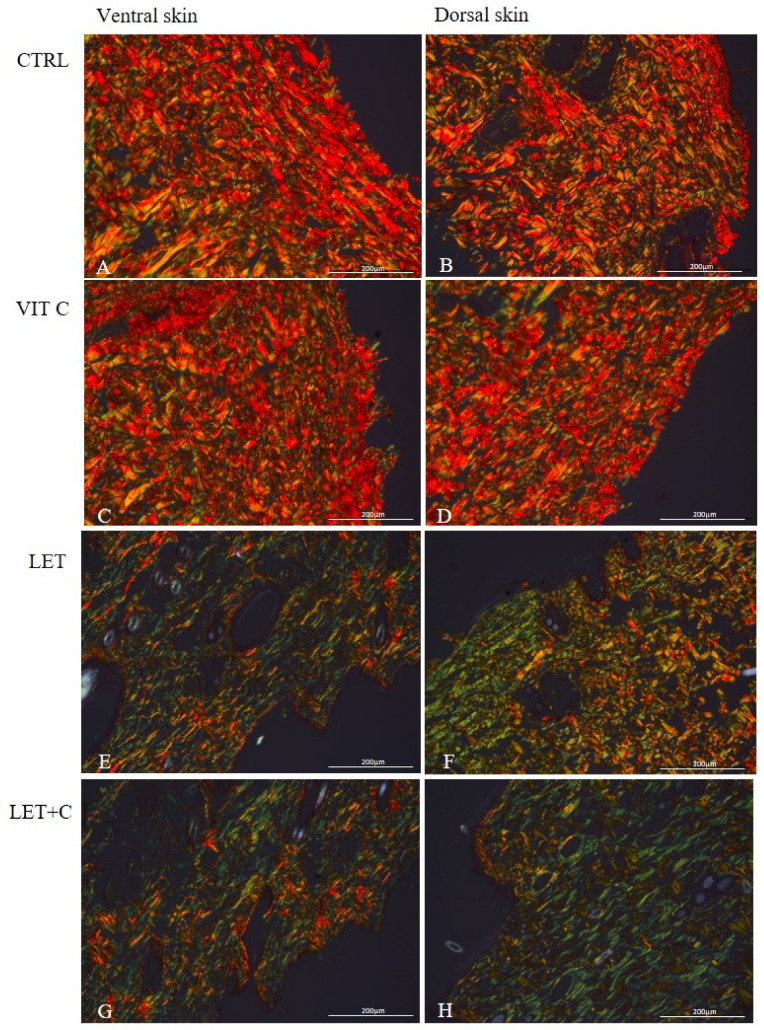
Collagen fibers in the ventral and dorsal skin. (**A**,**B**)—control group (CTRL); (**C**,**D**)—group supplemented with vitamin C (VIT C); (**E**,**F**)—letrozole-treated group (LET); (**G**,**H**)—letrozole-treated and vitamin C-supplemented group (LET+C); Picrosirius Red, objective magnification ×20.

After staining with Picrosirius Red, in the ventral and dorsal skin sections of the control group (Figure 2A,B) and the group supplemented with vitamin C (Figure 2C,D), a predominance of red color was observed, suggesting that the collagen fibers are thick and tightly packed. In the ventral and dorsal skin sections of the letrozole-treated group (Figure 2E,F) and the group simultaneously treated with letrozole and supplemented with vitamin C (Figure 2G,H), a lower intensity of red and yellow colors was observed, and a greater number of green fibers were observed, suggesting the presence of an increased number of altered and less packed fibers.

### 2.3. Morphometry

**Figure 3:**
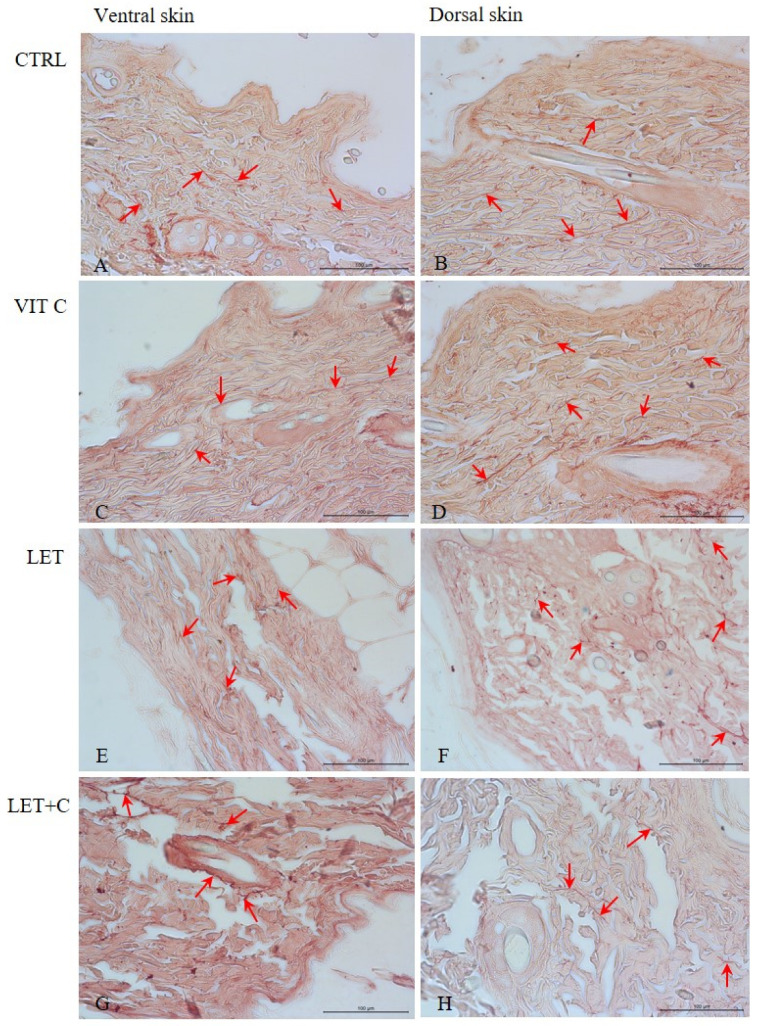
Organization of elastic fibers (red arrows) in the ventral and dorsal dermis. (**A**,**B**)—control group (CTRL); (**C**,**D**)—group supplemented with vitamin C (VIT C); (**E**,**F**)—letrozole-treated group (LET); (**G**,**H**)—letrozole-treated and vitamin C-supplemented group (LET+C). Orcein, objective magnification ×40.

In the ventral and dorsal skin sections of male rats from the CTRL (Figure 3A,B), VIT C (Figure 3C,D), LET (Figure 3E,F), and LET+C (Figure 3G,H) groups, thin and dark elastic fibers were observed interwoven with collagen fibers, mainly in the reticular layer of the skin. No statistically significant changes in the number of elastic fibers were observed in the dorsal and ventral skin between the CTRL and VIT C groups, between the CTRL and LET groups, and between the LET and LET+C groups (Table 1).

**Figure 4:**
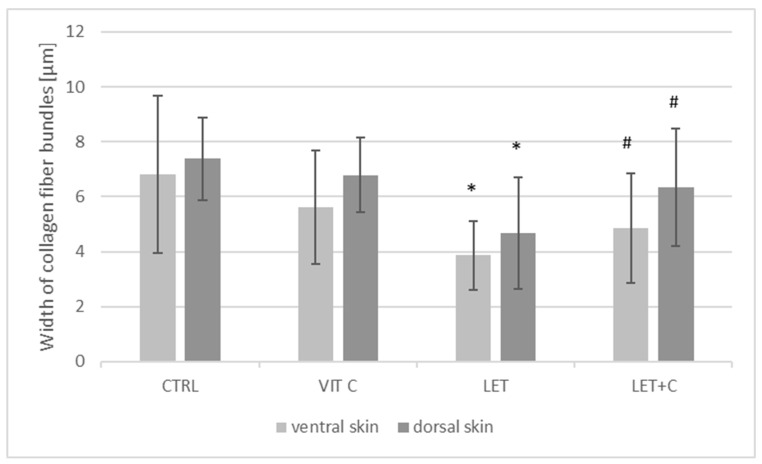
Width of collagen fiber bundles; mean ± SD; * *p* < 0.05 vs. CTRL, # *p* < 0.05 vs. LET.

No statistically significant changes in the width of collagen fiber bundles in the dorsal and ventral (Figure 4) skin were observed between the CTRL and VIT C groups. However, a statistically significant decrease in the dorsal and ventral skin was observed between the LET and CTRL groups. A statistically significant increase in the dorsal and ventral skin was observed in the LET+C group compared to the LET group (Table 1).

### 2.4. IHC Staining and Digital Analysis

Statistically significant differences were observed in the immunoexpression of catalase between the VIT C and CTRL groups and between the LET+C and LET groups in epidermal cells of dorsal skin, as well as between the VIT C and CTRL, LET and CTRL, and LET+C and LET groups in epidermal cells of ventral skin.

Statistically significant differences were observed in the immunoexpression of caspase-3 between the VIT C and CTRL groups in epidermal cells of dorsal skin, as well as between the LET and CTRL groups and between the LET+C and LET groups in epidermal cells of ventral skin.

**Figure 5:**
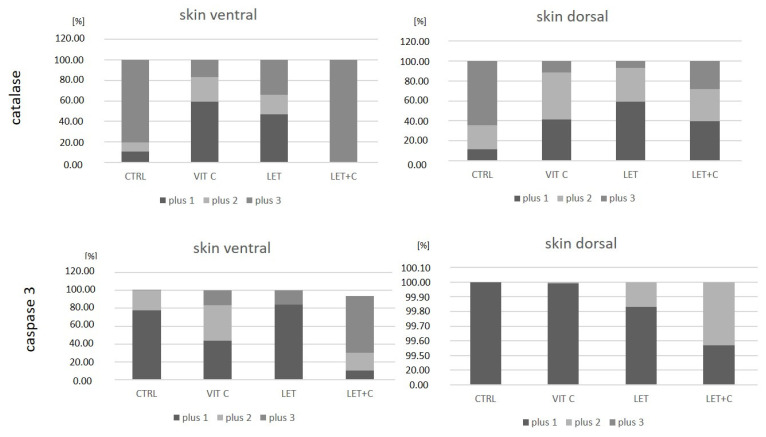
Percentage of immunopositive epidermal cells. Each bar represents 100% immunopositive cells in the group. The colors within each bar represent the percentage of immunohistochemistry intensity: +1—weak intensity; +2—medium intensity; and +3—strong intensity. The graphs were created using the 3DHISTECH Pannoramic MIDI II 2.5.0.143918 scanner (Sysmex Polska Sp. z o.o., Warsaw, Poland) software algorithm.

Additionally, the obtained results were calculated as the percentage of immunopositive cells and are presented as bar graphs (Figure 5 and Table 2). Spearman’s correlation analyses were performed to assess the associations between differences in the strength of protein immunostaining (Table 2).

## 3. Discussion

There are many studies on the role of estrogens in systems such as the nervous system, circulatory system, skeletal system, and skin. Estrogens exert their biological effect through estrogen receptors, which are widely distributed in skin tissues [35]. Estrogens affect important skin functions, such as hair growth, pigmentation, vascularity, elasticity, and water retention. The effect of estrogen replacement therapy on the skin and the ability of these hormones to improve wound healing in postmenopausal women [36,37] and delay the aging process [35] are very well documented. Estrogen alleviates mitochondrial dysfunction, which has been linked to the deterioration of skin with age [38]. Kovács et al. [39] and Singh and Paramanik [40] also suggest that estrogens and phytoestrogens affect epigenetic modifications, thereby maintaining genome integrity and reducing the accumulation of age-related changes. Furthermore, estrogens promote the maintenance of telomere length by regulating telomerase activity, thereby inhibiting cellular aging and, consequently, preserving cellular function and promoting skin rejuvenation [41,42]. Estrogens improve the condition of the skin in many ways, because they increase collagen content, have a positive effect on skin thickness and the number of wrinkles, and improve its hydration. They also affect skin appendages, such as hair [43].

Letrozole is a nonsteroidal aromatase inhibitor, belonging to the third generation of inhibitors. It blocks estrogen synthesis by inhibiting the last step of the estrogen biosynthesis pathway and is used in the treatment of disorders related to infertility [44]. Although it solves some problems related to male infertility, it can also affect skin morphology. We did not observe morphological changes in abdominal and dorsal skin sections between the CTRL and VIT C groups; however, we showed the presence of empty spaces between the fibers in the LET and LET+C groups. Similar results were obtained in the studies of Misiakiewicz-Has et al. [45]. According to Zouloboulis and Makrantonaki [46], a reduction in the extracellular matrix components of the dermis results in a change in the morphology of the dermal tissue. It is worth noting here that we also observed a statistically significant decrease in the width of collagen fiber bundles in the dorsal and ventral skin between the LET and CTRL groups. According to Markiewicz et al. [47], estrogens may not be directly involved in the regulation of collagen synthesis but may play an important role in regulating the organization and stability of collagen fibrils.

Staining with the Picrosirius Red method allows for the assessment of the organization of the extracellular matrix based on the birefringence of collagen fibers [48]. Collagen fibers are seen in shades of green, yellow, and red under a polarizing microscope. The color of polarization indicates the thickness and degree of fiber packing [49,50]. Thick and tightly packed collagen fibers are yellow to red, while collagen fibers showing a greenish color are disorganized, altered, and less packed [50]. In the ventral and dorsal skin sections of the animals from the CTRL and VIT C groups, we observed a predominance of red color, which suggests that the collagen fibers are thick and tightly packed [50]. However, in the ventral and dorsal skin parts of animals from the LET and LET+C groups, we observed a lower intensity of red and yellow colors and a higher content of green fibers. This suggests the presence of an increased amount of altered and less packed collagen fibers in the groups we studied [50], as well as the consequent deterioration of skin quality. Indeed, according to Reilly and Lozano [51], the quality of collagen fibers determines the quality of the whole skin. Similar results in their work were also obtained by Misiakiewicz-Has et al. [45]. Despite the small amount of direct research on letrozole and skin aging, two important links can be seen. The first is due to letrozole’s mechanism of action as an aromatase inhibitor, causing a reduction in estrogen levels, which is a documented factor in accelerating skin changes [52].

Data from in vitro cell culture studies have shown that one of the main factors promoting collagen synthesis in the skin is vitamin C. The proper functioning of fibroblasts depends on their presence. In addition, vitamin C supplementation acts as a cofactor for proline and lysine hydroxylases, which help stabilize the tertiary structure of the collagen molecule and promote the expression of collagen genes [53]. This can be seen as the reason for the increase in the width of collagen fiber bundles in the dorsal and ventral skin in the LET+C group compared to the LET group we observed. According to Gref et al. [54], vitamin C supply promotes the production of type III collagen in human skin and also mobilizes the production of glycosaminoglycans. The stability of the collagen that is synthesized varies depending on the amount of vitamin C available, illustrating the role of collagen bonds that are formed by hydroxylases [53].

Previous studies conducted at the Histology and Embryology Department have shown that the hormonal imbalance resulting from the long-term administration of letrozole to male rats led to pronounced morphological changes in many tissues and organs, similar to changes that occur during the aging process. In the case of skin, the symptoms described in the literature include thinning of the epidermis, slower wound healing, a reduced number of fibroblasts, a reduced volume of subcutaneous tissue, and a reduced number and diameter of collagen fibers [55]. Related anti-aging mechanisms within the skin, among others, include scavenging oxygen-free radicals and enhancing antioxidant defenses [56]. Free radicals disrupt collagen synthesis, degrade collagen and elastic fibers, and have a destructive effect on cell membrane lipids. This results in increased water loss and the occurrence of inflammation [57].

We observed a statistically significant increase in the immunoexpression of catalase between the VIT C and CTRL groups. As an electron donor, vitamin C can reduce reactive oxygen species and regenerate other antioxidants [58]. Studies [53,59] have shown that vitamin C increases catalase activity in keratinocytes and skin fibroblasts, which enhances protection against oxidative damage and promotes regenerative processes. Some antioxidants can inhibit catalase [60], while in animal and human models, vitamin C supplementation often correlates with increased activity of antioxidant enzymes, including catalase. Vitamin C mainly functions as an antioxidant. Yet, in specific situations involving free (unbound) redox metals like iron or copper, which arise from metal imbalance, vitamin C can display prooxidant characteristics [61]. Vitamin C increases catalase activity in the liver of rats with diabetes [62]. An increase in catalase expression was observed between the LET and CTRL groups, and the difference was statistically significant in the ventral skin. It can be assumed that the administration of an aromatase inhibitor may have become a stressor [63]. Estrogens bind to the ERs (ERα and ERβ), exerting their genomic and non-genomic effects. In the genomic pathway, estradiol binds to intracellular ERα and ERβ, forming dimers that then enter the cell nucleus and interact with DNA regulatory sequences (ERE, Ap1, and Sp1) to regulate gene transcription. The products of these genes affect processes such as autophagy, proliferation, apoptosis, survival, differentiation, and vasodilation. This effect develops slowly, within hours. In the non-genomic pathway, estradiol binds to membrane receptors (Erα and ERβ), leading to rapid activation of transcription factors through signaling pathways, including Ca^2+^ mobilization, phosphatidylinositol 3-kinase (PI3K), and mitogen-activated protein kinase (MAPK) activation. This action occurs within seconds to minutes and does not require direct transcriptional regulation [64]. Estrogen deficiency affects catalase expression through the modulation of pathways related to oxidative stress and the regulation of genes encoding the enzyme [65]. The administration of vitamin C and letrozole resulted in a decrease in catalase expression compared to the letrozole-treated group. Vitamin C, being a potent antioxidant, can alleviate side effects associated with taking aromatase inhibitors, such as oxidative stress and inflammation [66]. However, it is important to note that high doses of vitamin C can affect drug metabolism by interacting with liver enzymes [67], which, in turn, could theoretically alter the effectiveness of aromatase inhibitors. It is worth noting that the effect of vitamin C on catalase activity may vary depending on the chosen research model and experimental conditions. The effect of vitamin C depends not only on its dose but also on the condition of the skin and the presence of other stress factors [68].

It is also worth considering whether high-dose vitamin C may interact with letrozole pharmacodynamics or metabolism. There is a lack of direct clinical evidence that mechanisms correlated with high-dose vitamin C alter letrozole concentration. According to Shumyantseva et al. [69], vitamin C administered in high doses has pro-oxidative properties and may affect the activity of CYP3A4, an enzyme that metabolizes letrozole in the liver. In turn, CYP3A4 modulators may affect drug transporters [70], which may constitute an indirect pathway in the interaction between vitamin C and letrozole. However, further clinical studies are needed to fully understand the actual impact of vitamin C on the functioning of enzyme modulators and drug transporters and, consequently, on the efficacy or toxicity of drugs.

Estrogens affect various cellular processes, including apoptosis. Their effects depend on the specific tissue, the receptor type, and the level of exposure to these hormones [71]. This fact can be seen as an explanation for why we noted a decrease in caspase-3 expression in the ventral skin of the male LET group. Pang et al. [72] have previously explored the downstream signaling pathways of PI3K/Akt and established the paths by which estrogen protects against apoptosis. Estrogens regulate the activity of the PRODH/POX enzyme, which plays a key role in controlling apoptosis and autophagy. A decrease in estrogen concentration disrupts this pathway, promoting cell death [73]. The use of aromatase inhibitors could then contribute to skin cells’ susceptibility to apoptosis. Vitamin C neutralizes reactive oxygen species (ROS), which induce apoptosis and activate caspase 3. By reducing oxidative stress, vitamin C stabilizes the mitochondrial membrane, preventing the release of cytochrome c, and inhibits the cascade of caspase 3 activation. This protects cells from apoptosis [74]. For the vitamin C-supplemented groups, we noted an increase in caspase-3 expression in the VIT C group in dorsal skin and the LET+C group in ventral skin. It should be noted here that caspase-3 is a key enzyme in the process of apoptosis and also has functions unrelated to programmed cell death. According to Raymond AA et al. [75], caspase-3 displays clear expression in the epidermis, which may be involved in keratinocyte differentiation.

In conclusion, the results presented in this paper demonstrate that the administration of letrozole to male rats can adversely affect skin morphology in both the ventral and dorsal regions. Vitamin C supplementation, with the simultaneous administration of an aromatase inhibitor, may have a protective effect, but not in all cases. The results of this study underscore the need to use aromatase inhibitors with caution and to monitor patients for potential side effects. It is worth noting, however, that direct studies on the effects of high doses of vitamin C on the metabolism of aromatase inhibitors are limited. Therefore, although there are some suggestions of potential interactions, further studies are needed to determine exactly how vitamin C affects the efficacy of these drugs. The preclinical studies we present play an important role in understanding the basic mechanisms of interaction between vitamin C and aromatase inhibitors. Further clinical studies are required with appropriate dose adjustment, taking into account pharmacokinetics and pharmacodynamics in humans, as well as differences in vitamin C and letrozole metabolism between species. However, the experiments conducted on our model provide an opportunity for a preliminary assessment of the toxicity and safety of letrozole with simultaneous vitamin C supplementation by allowing the observation of potential adverse effects. This study may provide information on the effects of these substances at the cellular and molecular levels, which provides a basis for further clinical studies.

## 4. Materials and Methods

### 4.1. Animals and Tissues

The experiment was conducted in full accordance with Polish Law and with the approval of the ethics committee of the Pomeranian Medical University in Szczecin (approval no. 23/2017). Sexually mature 3-month-old male Wistar rats were randomly divided into four groups, as shown in Table 3: control (CTRL), vitamin C-supplemented (VIT C), letrozole (LET), and letrozole supplemented with vitamin C (LET+C). Each group consisted of 6 rats. Rats in the LET group received letrozole, a nonsteroidal inhibitor of cytochrome P450 aromatase, orally at a dose of 1 mg/kg b.w./day for 6 months (Femara, Novartis, Basel, Switzerland). LET was given to each experimental rat once per day in the morning in the form of a small pellet made from LET powder and pressed into a piece of bread. The animals willingly ate the pellets from the hand of the person experimenting. The rats in the CTRL group received a pellet without LET with a piece of bread. The rats in the VIT C group received water with vitamin C (Ascorgem, Karczew, Poland) at a dose of 500 mg/L. The method of vitamin C administration was taken from the literature and is based on the average daily fluid intake by rats. The rats in the LET+C group received a pellet with LET pressed into a piece of bread and water supplemented with vitamin C (Table 3). The animals were treated for 6 months. At the end of the experimental treatment, the animals were weighed (Table 3) and sacrificed under thiopental anesthesia following the approved protocol. The dorsal and ventral skins were collected, fixed in formalin, and embedded in paraffin. A series of sections (3–5 µm) was prepared from the paraffin-embedded tissues.

### 4.2. Staining, Morphology, and Morphometry

For the morphological and morphometrical analyses, dewaxed and hydrated sections were stained with hematoxylin–eosin, orcein, and Sirius Red methods using the manufacturer’s protocol. Histological measurements were made using the LAS 1.15.0.46.43 imaging analysis software under a light Leica microscope (Leica DM5000B, Wetzlar, Germany).

Collagen fiber width measurements (average of 150 for each group) were performed on ventral and dorsal skin sections stained with the HE method. The measured collagen fiber bundles were located in the reticular layer of the dermis. Morphometric measurements were performed at ×20 magnification using the LAS V4.4 program.

The assessment of the number of elastic fibers was performed on cross-sections of the ventral and dorsal skin (average of 60 for each group), stained with orcein. The analyzed elastic fibers were located in the papillary and reticular layers of the dermis. The analysis was performed at ×20 magnification, using the FIJI ImageJ 1.8.0_172 program, and presented as a percentage of the area occupied by these fibers.

### 4.3. IHC Staining

To identify the parameters of programmed cell death markers and oxidative damage, specific antibodies and immunohistochemistry reactions were employed using the EnVision system (Agilent, Santa Clara, CA, USA). The slides were incubated with primary antibodies at room temperature for 1 h. The antibody used to identify oxidative damage was mouse monoclonal anti-catalase antibody, Santa Cruz Biotechnology (Dallas, TX, USA), cat no:sc-365738; dilution 1:200. The antibody used to identify apoptotic cells was mouse monoclonal anti-caspase-3 antibody, Santa Cruz Biotechnology (Dallas, TX, USA), cat no:sc-56053; dilution 1:200. The antibodies were diluted in Antibody Diluent (cat# ab64211, abcam, Cambridge, UK). After washing, the slides were covered with ready-to-use EnVision FLEX LINKER for 30 min at RT. To visualize the antigen–antibody complex, a reaction of avidin–biotin–horseradish peroxidase with DAB as a chromogen was performed, following the included staining procedure instructions. The sections were washed in distilled H_2_O and counter-stained with hematoxylin. Negative controls were processed without the primary antibody. Positive and negative stainings were determined by visual identification of brown pigmentation using a microscope (Leica DM5000B, Wetzlar, Germany).

### 4.4. Digital Analysis

**Figure 6:**
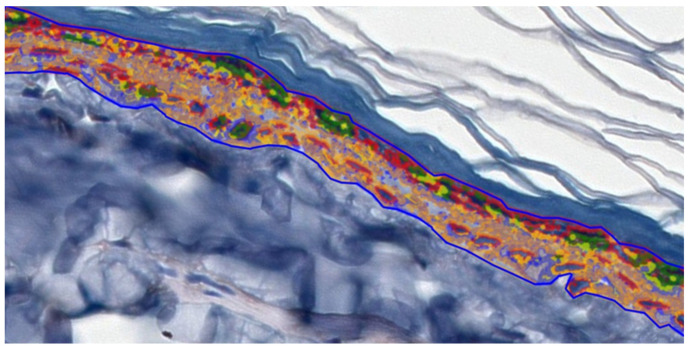
Quantitative digital image analysis created by Pattern Quant. Red circles—cell nuclei; green circles—+3 cytoplasm intensity; yellow circles—+2 cytoplasm intensity; blue circles—+1 cytoplasm intensity; and orange circles—negative.

To facilitate digital image analysis, IHC-stained slides were scanned using a 3DHISTECH Pannoramic MIDI II scanner (Sysmex Polska Sp., Warsaw, Poland), with a lens magnification of 20×, resulting in images with a resolution of 0.17 µm/pixel. The analysis was performed in the epidermis, excluding the stratum corneum. Ten epidermal areas in which the basement membrane length was 200 µm were selected. The Pattern Quant 2.3 software from 3DHISTECH (3DHISTECH Kft., Budapest, Hungary) was used for digital image analysis. Quantitative digital image analysis was employed to assess epidermal IHC staining. Parameters such as area, perimeter occupied by the assessed structure, and the intensity of staining were determined. Since Pattern Quant is software that visualizes different structures based on (i) pattern and (ii) color, we were able to examine cell nuclei and cytoplasm. We trained the program to recognize the tested structures. We divided the separated structures into the following clusters: red circles—cell nuclei; green circles—+3 strong cytoplasm intensity; yellow circles—+2 medium cytoplasm intensity; blue circles—+1 weak cytoplasm intensity; and orange circles—negative (Figure 6). The results obtained by the Pattern Quant were expressed as values (area). Additionally, the obtained results were calculated as a percentage of cell nuclei and cytoplasm and are presented as bar graphs. Two independent experts analyzed all calculations.

### 4.5. Statistical Analysis

The results were analyzed statistically using Statistica 6.1 software (StatSoft, Kraków, Poland). In the first part of the analysis, the normality of the distribution of the obtained data was checked using the Shapiro–Wilk test. Since the distribution of at least one of the groups deviated from the normal distribution, the Kruskal–Wallis ANOVA test was used. A multiple comparison post hoc test was employed to evaluate the differences between the studied groups. Medians, arithmetical means, and standard deviations (±SD) were determined for each parameter. Spearman’s correlation analyses were performed to assess associations between differences in the strength of protein immunoexpression.

## Figures and Tables

**Table 1:** Summary of the morphometric measurements and immunohistochemical analysis comparing data from all experimental groups.

	Groups			
	CTRL	VIT C	LET	LET+C
Elastic fibers [%]				
Dorsal				
X ± SD	0.38 ± 0.37	0.30 ± 0.20	0.15 ± 0.05	0.17 ± 0.13
Me	0.15	0.23	0.15	0.15
				
Ventral				
X ± SD	0.21 ± 0.12	0.19 ± 0.09	0.21 ± 0.16	0.34 ± 0.10
Me	0.16	0.19	0.16	0.39
Collagen fibers [µm]				
Dorsal				
X ± SD	7.38 ± 1.51	6.79 ± 1.35	4.68 ± 2.04	6.33 ± 2.14
Me	6.83	6.54	4.27 *	6.21 ^#^
				
Ventral				
X ± SD	6.82 ± 2.87	5.61 ± 2.08	3.86 ± 1.26	4.84 ± 1.99
Me	6.19	4.97	3.77 *	4.55 ^#^
Catalase [µm^2^]				
Dorsal				
X ± SD	102.85 ± 145.96	772.76 ± 286.18	554.12 ± 251.59	11.03 ± 21.19
Me	29.40	767.7 *	441.39	0.88 ^#^
				
Ventral				
X ± SD	196.18 ± 146.86	626.84 ± 133.57	650.14 ± 550.99	196.34 ± 144.37
Me	149.25	594.85 *	488.87 *	145.97 ^#^
Caspase-3 [µm^2^]				
Dorsal				
X ± SD	48.79 ± 51.16	277.48 ± 76.46	114.25 ± 91.64	377.40 ± 315.65
Me	32.85	291.15 *	71.94	235.00
				
Ventral				
X ± SD	464.30 ± 187.87	398.21 ± 175.58	27.09 ± 27.30	563.57 ± 351.82
Me	412.85	348.78	21.87 *	503.76 ^#^

CTRL—control group; VIT C—group supplemented with vitamin C; LET—letrozole-treated group; LET+C—letrozole-treated and vitamin C-supplemented group; Me—median; X—mean; SD standard deviation; * *p* < 0.05 vs. CTRL, # *p* < 0.05 vs. LET.

**Table 2:** Spearman’s correlation table for the percentage of immunopositive epithelial cells, categorized by the strength of immunostaining.

	Catalase		Caspase-3	
	r_s_		r_s_	
	Ventral Skin	Dorsal Skin	Ventral Skin	Dorsal Skin
VIT C:CTRL	−0.50000	−0.50000	1.00000	0.866025
LET:CTRL	0.500000	−1.00000	0.500000	0.866025
LET:LET+C	−0.500000	1.00000	−0.50000	1.00000

CTRL—control group; VIT C—group supplemented with vitamin C; LET—letrozole-treated group; LET+C—letrozole-treated and vitamin C-supplemented group; r_s_—Spearman’s correlation; *p* < 0.05000.

**Table 3:** Experimental groups. The treatment lasted 6 months. n = 6.

No.	Group	Description	Treatment	Body Weight [g]
1.	CTRL	Control group	A bread pellet	500.83
2.	VIT C	Group supplemented with vitamin C	A bread pellet + vitamin C, at a dose of 500 mg/L of water	563.33
3.	LET	Letrozole-treated group	A bread pellet with LET at a dose of 1 mg/kg b. w./day	439.17
4.	LET+C	Letrozole-treated and vitamin C-supplemented group	A pellet with LET at a dose of 1 mg/kg b. w./day + water with vitamin C at a dose of 500 mg/L	467.5

## Data Availability

The data presented in this study are available upon request from the corresponding author.

## Abbreviations

The following abbreviations are used in this manuscript:

P450arom	cytochrome P450 aromatase
ER	estrogen receptor
TGFβ	transforming growth factor-beta
TIMPs	tissue inhibitors of matrix metalloproteinases
MMPs	matrix metalloproteinases
LET	letrozole
CTRL	control
VIT C	control supplemented with vitamin C
LET+C	letrozole supplemented with vitamin C
HE	hematoxylin and eosin staining
Me	median
X	mean
SD	standard deviation
r_s_	Spearman’s correlation
PI3K	phosphatidylinositol 3-kinase
MAPK	mitogen-activated protein kinase
ROS	reactive oxygen species
